# Comprehensive Construction of a Circular RNA-Associated Competing Endogenous RNA Network Identified Novel Circular RNAs in Hypertrophic Cardiomyopathy by Integrated Analysis

**DOI:** 10.3389/fgene.2020.00764

**Published:** 2020-07-28

**Authors:** Qi Guo, Junjie Wang, Runlu Sun, Zhijian He, Qian Chen, Wenhao Liu, Maoxiong Wu, Jinlan Bao, Zhaoyu Liu, Jingfeng Wang, Yuling Zhang

**Affiliations:** ^1^Department of Cardiology, Sun Yat-sen Memorial Hospital of Sun Yat-sen University, Guangzhou, China; ^2^Guangdong Province Key Laboratory of Arrhythmia and Electrophysiology, Guangzhou, China

**Keywords:** hypertrophic cardiomyopathy, circular RNAs, competing endogenous RNA network, weighted correlation network analysis, microarray

## Abstract

Hypertrophic cardiomyopathy (HCM), the most common heritable cardiomyopathy, is associated with a high risk of sudden cardiac death. The complexity and behavior of the circular RNA (circRNA)-associated competing endogenous RNA (ceRNA) network in HCM have not been thoroughly elucidated. Plasma circRNA and messenger RNA (mRNA) expression profiles were acquired by using a microarray. Weighted correlation network analysis (WGCNA) and linear models for microarray data (Limma) were used to analyze microarray data. Gene modules, consisting of genes with high correlations, were detected and represented by a designated color. The ceRNA network, including circRNA, microRNA (miRNA), and mRNA, was constructed based on the “ceRNA hypothesis” using an integrated systems biology method. By WGCNA, two modules, namely magenta and red modules, were identified as being positively correlated with HCM. In the combined analysis of WGCNA and Limma, 36 hub circRNAs in the magenta module and 83 hub circRNAs in the red module were significantly upregulated compared with the controls. By coexpression analysis, 270 circRNA–mRNA pairs were identified with a coefficient ≥0.9 and *p* < 0.05. With Starbase and miRWalk tools, circRNA–miRNA pairs and miRNA–mRNA pairs were predicted. Once these pairs were combined, the ceRNA network with 6 circRNAs, 29 miRNAs, and 6 mRNAs was constructed. Functional analysis demonstrated that these circRNAs in the ceRNA network were associated with calcium-release channel activity and muscle filament sliding. Our study provided a global perspective and systematic analysis of the circRNA-associated ceRNA network in HCM. The identified circRNAs hsa_circ_0043762, hsa_circ_0036248, and hsa_circ_0071269 may be key regulators involved in HCM pathogenesis.

## Introduction

Hypertrophic cardiomyopathy (HCM) is widely recognized as an autosomal dominantly inherited cardiac disorder, and its prevalence has increased considerably with the popularization of genomic sequencing (Elliott et al., 2014). In recent decades, the discovery of HCM-causing mutations has contributed to a strong improvement in the management of risk for adverse outcomes, such as sudden cardiac death, heart failure, atrial fibrillation, and non-fatal stroke (Olivotto et al., 2008). However, it has been found that HCM-causing sarcomere mutations are absent in approximately 70% of patients with established disease, and sarcomere gene carriers can live to advanced ages without developing HCM (Maron et al., 2019). It is of considerable importance to expand the HCM research focus beyond a single molecular event toward more inclusive models, including transcriptional or epigenetic factors, to explain this disease in its entirety.

Circular RNAs (circRNAs) are a class of closed circular non-coding RNAs. The lack of free ends on circRNAs confers increased stability compared with linear transcripts, making them ideal candidates for diagnostic biomarkers and therapeutic interventions (Barrett and Salzman, 2016). Accumulating evidence has shown crucial regulatory functions of circRNAs in numerous cardiovascular diseases, such as atherosclerosis, myocardial infarction, and heart failure (Altesha et al., 2019; Van Heesch et al., 2019; Gomes et al., 2020). Moreover, several studies have indicated circRNAs with cardiac hypertrophy and fibrosis, both of which are important pathological changes in HCM (Wang et al., 2016; Yousefi and Soltani, 2020). Thus, it is hypothesized that circRNAs may also play vital roles in the pathogenesis of HCM.

Over the past decade, the competing endogenous RNA (ceRNA) hypothesis has attracted attention from researchers. According to this hypothesis, in addition to microRNAs (miRNAs), multiple molecules also participate in posttranscriptional regulatory networks, such as long non-coding RNAs, cirRNAs, and some pseudogenes, which can similarly function as ceRNAs to suppress miRNAs and interfere with mRNAs. Previous research demonstrated that in thoracic aortic constriction and isoproterenol−induced cardiac hypertrophy models, the expression of heart−related circRNA was reduced, whereas miR−223 was increased, compared with sham or non-failing control hearts (Werfel et al., 2016). In terms of pressure overload-induced hypertrophy, a highly abundant circRNA named *circSlc8a1* was reported to function as an endogenous sponge for miR-133a in cardiomyocytes and therefore attenuate pressure overload-induced hypertrophy (Lim et al., 2019). However, the expression profile of circRNAs and the complexity of the circRNA-associated ceRNA network in the pathogenesis of HCM have not been thoroughly characterized to date.

To investigate the regulatory mechanism of circRNAs in HCM, especially the complex interactions among circRNAs, miRNAs, and mRNAs, we collected plasma samples from HCM patients and healthy controls and profiled their circRNA expression with a microarray. The hub differentially expressed circRNAs were identified by the method of linear models for microarray data (Limma) and weighted correlation network analysis (WGCNA), and the biological functions of their interaction miRNAs and mRNAs were analyzed (Langfelder and Horvath, 2008; Ritchie et al., 2015). Our results might help to elucidate the roles of circRNA in HCM and propose possible interaction mechanisms among circRNAs, miRNAs, and mRNAs.

## Materials and Methods

### Study Population

The study population consisted of 15 HCM patients and 7 healthy control subjects at Sun Yat-sen Memorial Hospital of Sun Yat-sen University from October 2018 to May 2019. According to the European Society of Cardiology Guidelines, HCM in adults was defined by a wall thickness =15 mm in one or more left ventricular myocardial segments or a wall thickness =13 mm in one or more left ventricular myocardial segments with HCM history in first-degree relatives, as measured by echocardiography or cardiac magnetic resonance (Elliott et al., 2014). The exclusion criteria were as follows: a history of ventricular septal surgery, valvular heart disease, coronary artery disease, atrial fibrillation, systemic hypertension, diabetes, surgery, or trauma within 6 months, cancer, and renal dysfunction.

This study was carried out following the recommendations of the ethics committee of Sun Yat-sen Memorial Hospital, and all subjects provided written informed consent following the Declaration of Helsinki.

### Plasma Collection and Microarray

Whole blood was collected in venous blood collection tubes containing ethylenediaminetetraacetic acid after fasting overnight, and plasma was separated by centrifugation. Total RNA was isolated using a Plasma RNA Purification Mini Kit (Norgen, Thorold, Canada) according to the manufacturer’s instructions and purified using an RNeasy Mini Kit (Qiagen, GmBH, Germany). Total RNA was examined for RNA integrity number to inspect RNA integration by an Agilent Bioanalyzer 2100 (Agilent Technologies, Santa Clara, United States). RNA samples from each group were then used to generate biotinylated complementary RNA targets for the LC Human ceRNA array (version 1.0). The biotinylated complementary RNA targets were then hybridized with the slides. After hybridization, slides were scanned on an Agilent Microarray Scanner (Agilent Technologies, Santa Clara, United States). The microarray experiments were performed by following the protocol of Agilent Technologies Inc. at the LC Sciences Corporation. All microarray data have been uploaded to the Gene Expression Omnibus database with the identification of GSE148602.

**FIGURE 1:**
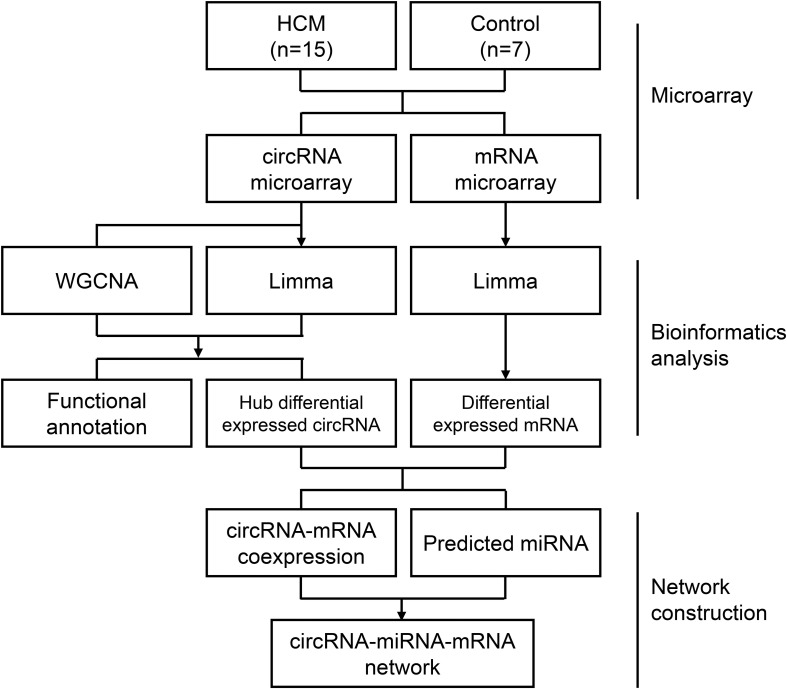
Flow chart of the approach utilized in the present study. The WGCNA R package was used to construct a weighted correlation network. Differentially expressed RNAs was identified by Limma package in R. Correlation coefficients, miRWalk database, and Starbase database were enrolled for ceRNA network construction. HCM, hypertrophic cardiomyopathy; circRNA, circular RNA; miRNA, microRNA; mRNA, messenger RNA; WGCNA, weighted gene coexpression network analysis; Limma, linear models for microarray data.

Raw data were extracted with Feature Extraction software (version 12.0.3.1, Agilent Technologies, Santa Clara, United States) (Supplementary Table S1). After data extraction from slides, raw data were normalized by the quantile algorithm using Genespring software (version 14.8, Agilent Technologies, Santa Clara, United States) (Supplementary Table S2). A quantile algorithm is a common normalization method that could transform each sample to have the same quartiles (Suyundikov et al., 2015). The distribution of RNAs in each sample was transformed to be the same after quantile normalization, which was showed in box plots (Supplementary Figure S1). In our study, biological replicates, including 15 HCM samples and 7 control samples, were used. The exclusion criteria for filtering out were that probes were detected in less than 20% samples of each group. After microarray data normalization and filtering, subsequent bioinformatic analysis and network construction were performed according to our flow chart (Figure 1).

### Weighted Correlation Network Analysis

The WGCNA R package was used to construct a weighted correlation network using circRNA expression data measured as discussed earlier (Langfelder and Horvath, 2008). In the network, the pairwise Pearson coefficient was calculated to evaluate the weighted coexpression relationship among all genes in the adjacency matrix. The soft threshold was used to ensure a scale-free network. The topological overlap measure was used to represent the network interconnectedness. Gene modules, consisting of genes with high correlations, were detected using the hierarchical clustering method based on a topological overlap measure-based dissimilarity measure (Ravasz et al., 2002). A designated color represented each module. The identification of hierarchical clustering dendrograms was conducted by using the dynamic branch cut method (Langfelder et al., 2008).

### Identification of Clinically Key Modules and Functional Annotation

Modules from hierarchical clustering with significant correlations with clinical traits were identified as key modules for further analysis. Gene significance, module significance, and module eigengene were calculated. Briefly, gene significance was defined as the negative log of a *p*-value, module significance was the average gene significance across the module gene, and module eigengene was the first principal component of a given module. As modules with a high trait significance could be highly associated with HCM, the significance between the ME and a clinical trait of HCM was also calculated (Fuller et al., 2007). Gene ontology (GO) and Kyoto Encyclopedia of Genes and Genomes (KEGG) functional annotations by ClueGO app in Cytoscape (Version 3.7.2, United States) were performed to further investigate the key modules by finding the potential function and underlying mechanism (Shannon et al., 2003; Bindea et al., 2009).

### Identification of Differentially Expressed Hub Circular RNAs

An absolute value of geneModuleMembership >0.8, geneTraitSignificance >0.2, and q.weighted <0.01 were used to screen the hub genes of the key module (Horvath and Dong, 2008). We identified differentially expressed circRNAs using the linear models for microarray data (Limma) package in R (Ritchie et al., 2015). All *p*-values were adjusted by the Benjamini–Hochberg method in Limma analysis.

### Construction of Competing Endogenous RNA Network

Based on the theory of ceRNA, circRNA and mRNA in the network shall share a similar expression trend. Thus, each of the hub differentially expressed circRNAs was compared with differentially expressed mRNAs one by one to calculate the correlation coefficient. Those circRNA–mRNA pairs with correlation efficiencies ≥0.9 and *p* < 0.05 were selected for further analysis. Targeted miRNAs by circRNAs were predicted using Starbase v2.0 (Li et al., 2014). Targeted miRNAs by mRNAs were predicted using miRWalk v2.0 with 12 algorithms (TargetScan, RNAhybrid, RNA22, PITA, Pictar2, miRWalk, Microt4, miRNAMap, miRDB, miRridge, miRanda, and miRMap) (Dweep and Gretz, 2015). Those miRNAs identified by more than seven algorithms were chosen. Then, the miRNA–circRNA and miRNA–mRNA interactions that shared at least one miRNA were retained to construct the circRNA–miRNA–mRNA network. The functional annotation of each circRNA in the network was analyzed with those differentially expressed mRNAs between the circRNA upregulated group and downregulated group.

### Statistical Analysis

Categorical parameters are presented as *n* (%), and continuous parameters are presented as the mean ± SD. Student’s *t*-test and Fisher’s exact probability test were used to compare differences between the HCM group and the control group. Differences were considered to be significant at *p* < 0.05 if not specified. All statistical analyses were performed with the R tool (version 3.6.1).

## Results

### Clinical Characteristics of Enrolled Subjects

A total of 15 HCM and 7 healthy controls were enrolled in our study, with an average age of 57.81 years. There was no significant difference in age, sex, or smoking status between the HCM and control groups (*p* > 0.05). Total cholesterol and white blood cell count were significantly higher, whereas the blood glucose level was significantly lower in the HCM group (*p* < 0.05). In terms of cardiac ultrasound indexes, the HCM group showed a significantly higher left ventricular diastolic dimension and left atrial diameter compared with the control group (*p* < 0.05) (Supplementary Table S3).

### Construction of Weighted Gene Coexpression Networks

**FIGURE 2:**
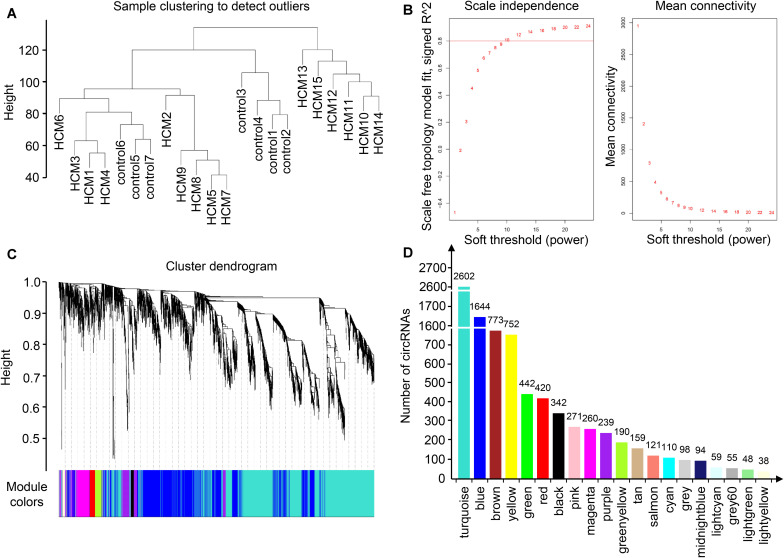
Identification of modules in HCM by WGCNA. **(A)** Sample clustering to detect outliers. Samples from HCM patients and controls were well distinguished in this dendrogram, indicating the difference of circRNA expression profile. **(B)** Analysis of network topology for various soft-thresholding powers. Each power corresponded to a scale independence and mean connectivity. A soft threshold of 10 (red horizontal line) was selected for construction of the scale-free topology module. **(C)** Clustering dendrograms of circRNAs. **(D)** The number of circRNAs in each module. One module was named with one color, and each color corresponded to a unique module. The number of circRNAs in each module was shown above each column. As a result, 20 coexpression modules were constructed. HCM, hypertrophic cardiomyopathy; WGCNA, weighted gene coexpression network analysis; circRNA, circular RNA.

To identify the relatively balanced scale independence and mean connectivity, a power value of 10 was selected for the construction of the scale-free topology module. A total of 20 coexpression modules were constructed, in which the turquoise module was the largest, containing 2,602 circRNAs, and the light-yellow module was the smallest, containing 38 circRNAs (Figure 2). Module interaction analysis showed that modules were independent of each other (Supplementary Figure S2).

### Identification and Functional Annotation of Key Modules Associated With Hypertrophic Cardiomyopathy

**FIGURE 3:**
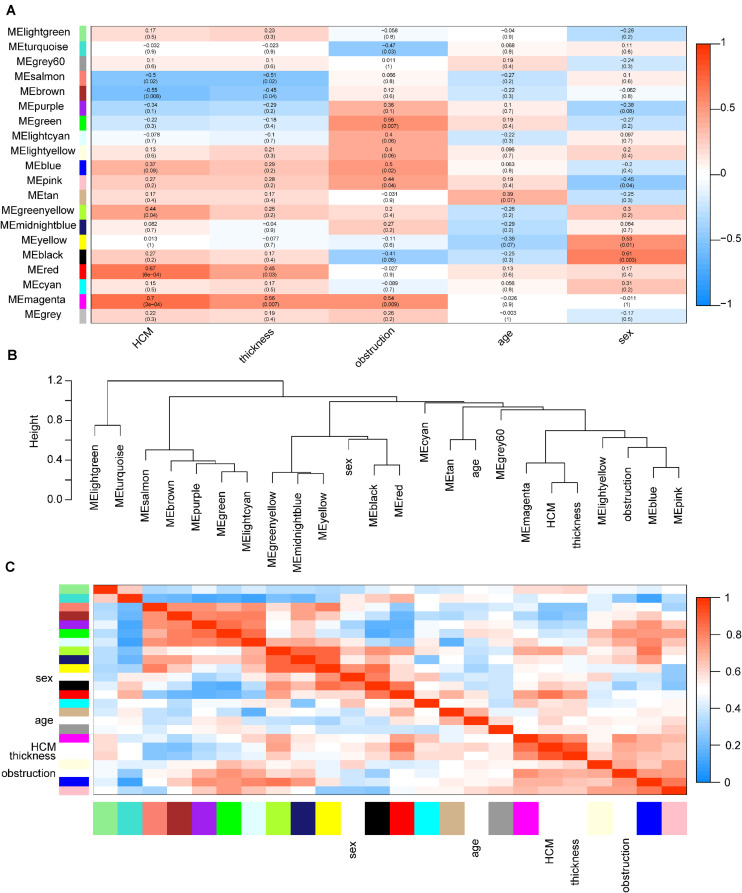
Identification of key modules in HCM. **(A)** Module-trait associations. Each row corresponds to a module eigengene, column to a trait. Each cell contains the corresponding correlation coefficient and *p*-value. The table is color coded by correlation according to the color legend. Red color indicated a positive correlation, and blue color indicated a negative correlation. The names of modules were showed in the left of the panel. The magenta and red modules showed the most significant association with HCM. **(B)** The eigengene dendrogram of correlated eigengenes. The dendrogram indicates that the magenta module is highly related to HCM and maximum left ventricular wall thickness. **(C)** Heatmap of modules associations. The heatmap in the panel shows the eigengene adjacency. Red color indicated a remarkable correlation between modules. The color blocks in the left and bottom of the panel corresponded to modules. HCM, hypertrophic cardiomyopathy; ME, module eigengene.

Among these modules, the magenta and red modules were positively associated with HCM with coefficients of 0.70 (*p* = 3e-4) and 0.67 (*p* = 6e-4), respectively (*p* < 0.05). The dendrogram and heatmap suggested that the magenta module was highly related to HCM and maximum left wall thickness (Figure 3). Thus, magenta and red modules were chosen as key modules for HCM. Based on intramodular analysis, high gene significance for HCM and module membership were characterized in the magenta (correlation coefficient = 0.56, *p* = 7.3e-23) and red modules (correlation coefficient = 0.51, *p* = 3.4e-29) (Supplementary Figure S3). In the magenta module, GO analysis showed that biological processes were enriched in, for instance, target of rapamycin signaling, protein modification, and vesicle cargo loading. KEGG pathway analysis demonstrated that these circRNAs might function through the thyroid hormone signaling pathway and transforming growth factor-beta signaling pathway. In the red module, GO analysis showed that biological processes were related to protein catabolic processes. KEGG pathway analysis demonstrated that these circRNAs might function through ubiquitin-mediated proteolysis and the mammalian target of rapamycin signaling pathway (Supplementary Figure S4).

### Identification of Hub Differentially Expressed Circular RNAs in Key Modules

By Limma, a total of 391 upregulated circRNAs, 384 downregulated circRNAs, 229 upregulated mRNAs, and 140 downregulated mRNAs were identified comparing the transcriptome expression profiles of HCMs and controls (Supplementary Figure S5). Under the criteria of gene significance >0.2, module membership value >0.8, and q.weighted <0.01, 47 hub circRNAs and 193 hub circRNAs were selected from the magenta module and red module, respectively. After an integrated analysis of differentially expressed data and hub circRNA data, 35 hub differentially expressed circRNAs in the magenta module and 83 hub differentially expressed circRNAs in the red module were identified (Supplementary Tables S4, S5).

### Competing Endogenous RNA Network Construction

**FIGURE 4:**
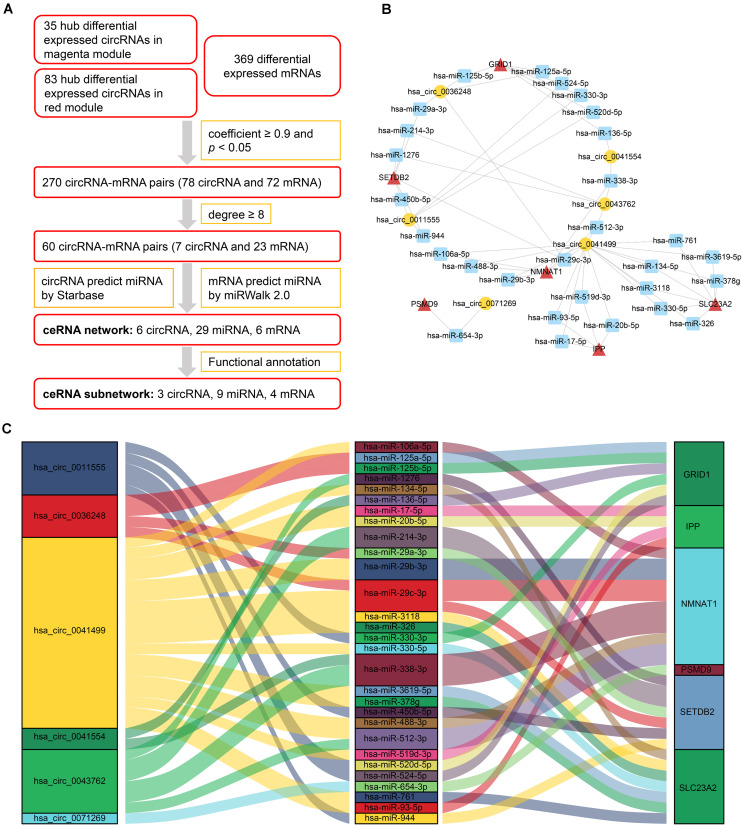
CeRNA network of circRNA–miRNA–mRNA in HCM. **(A)** Flow chart for ceRNA network construction. Correlation coefficients were used for screening of circRNA–mRNA pairs. Starbase and miRWalk were used to predict the potential interacting miRNAs. **(B)** CircRNA–miRNA–mRNA network. Circular block represented circRNAs, square block represented miRNA, and triangle block represented mRNA. A ceRNA network including 6 circRNAs, 29 miRNAs, and 6 mRNAs was constructed by integrated analysis. **(C)** Sankey diagram of circRNA-associated ceRNA network. Each color block corresponded to one RNA, and the link between two blocks indicated a potential interaction between two RNAs. The Sankey diagram clearly showed the potential interactions between 6 circRNAs and 29 miRNAs as well as interactions between 29 miRNAs and 6 mRNAs. HCM, hypertrophic cardiomyopathy; circRNA, circular RNA; miRNA, microRNA; mRNA, messenger RNA; ceRNA, competing endogenous RNA.

After analyzing correlation coefficients between 118 hub differentially expressed circRNAs and 369 differentially expressed mRNAs, 60 circRNA–mRNA pairs, including 7 circRNAs and 23 mRNAs, were selected based on a coefficient ≥0.9 and degree ≥8. To construct the circRNA–miRNA–mRNA network, circRNA–miRNA pairs and miRNA–mRNA pairs were predicted by Starbase and miRWalk tools. Once these pairs were combined, a preliminary ceRNA network with 6 circRNAs, 29 miRNAs, and 6 mRNAs was constructed. A Sankey diagram is a visualization used to depict links from one gene to another. The Sankey diagram clearly showed the potential interactions between 6 circRNAs and 29 miRNAs as well as interactions between 29 miRNAs and 6 mRNAs (Figure 4).

### Function of Competing Endogenous RNA Network-Associated Circular RNAs

**FIGURE 5:**
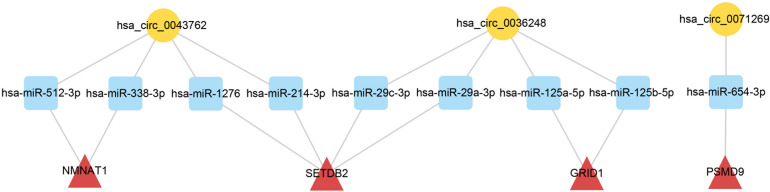
CeRNA subnetwork of circRNA–miRNA–mRNA in HCM. Circular block represented circRNAs, square block represented miRNA, and triangle block represented mRNA. After identification of three HCM-associated circRNAs, a ceRNA subnetwork originating from these three circRNAs was constructed by integrated analysis. This ceRNA subnetwork included 3 circRNAs, 9 miRNAs, and 4 mRNAs. HCM, hypertrophic cardiomyopathy; circRNA, circular RNA; miRNA, microRNA; mRNA, messenger RNA; ceRNA, competing endogenous RNA.

To elucidate the biological functions of ceRNA network-associated circRNAs, GO and KEGG analyses were performed. GO analysis revealed that hsa_circ_0071269 and hsa_circ_0043762 were enriched in calcium-release channel activity. In contrast, hsa_circ_0036248 was enriched in calcium-release channel activity and muscle filament sliding. KEGG analysis demonstrated that hsa_circ_0036248 might be associated with the regulation of transient receptor potential (TRP) channels, adrenergic signaling in cardiomyocytes, and calcium signaling pathways. hsa_circ_0071269 may be associated with the regulation of TRP channels and dilated cardiomyopathy (Supplementary Table S6). Then, a ceRNA subnetwork originating from these three circRNAs was constructed, which were predicted to be functionally related to HCM (Figure 5).

## Discussion

Hypertrophic cardiomyopathy is one of the most common hereditary heart diseases and is associated with a high risk of sudden cardiac death (Elliott et al., 2014). Over the past several decades, considerable efforts have been made to elucidate the molecular mechanisms underlying HCM, and the focus has been on protein-coding genes, most of which are responsible for sarcomere construction. Recently, circRNAs have been widely reported to participate in a wide range of biological processes, and their dysregulated expression is associated with many complicated human disease phenotypes, including cardiovascular diseases (Altesha et al., 2019).

In this study, plasma circRNA and mRNA expression profiles were acquired by using a microarray. By WGCNA, magenta and red modules were identified to be positively correlated with HCM. In the combined analysis of WGCNA and Limma, 36 hub circRNAs in the magenta module and 83 hub circRNAs in the red module were significantly upregulated compared with the controls. By coexpression analysis, 270 circRNA–mRNA pairs were identified with a coefficient ≥ 0.9 and *p* < 0.05. To construct the circRNA–miRNA–mRNA network, circRNA–miRNA pairs and miRNA–mRNA pairs were predicted by Starbase and miRWalk tools. Once these pairs were combined, the ceRNA network with 6 circRNAs, 29 miRNAs, and 6 mRNAs was constructed. Functional analysis demonstrated that these circRNAs in the ceRNA network were associated with calcium-release channel activity and muscle filament sliding. To the best of our knowledge, this study is the first to provide a global view and systematic dissection of the circRNA-associated ceRNA network in HCM.

CircRNAs are endogenous transcripts with multiple miRNA response elements, suggesting that they can interact with the miRNA seed region to alter miRNA activity in the manner of ceRNA (Memczak et al., 2013). Once hub differentially expressed circRNAs and mRNAs were identified, potential common target miRNAs were predicted by Starbase and miRWalk tools. Then, a circRNA–miRNA–mRNA network in HCM was constructed according to ceRNA theory. A previous study found that hsa_circ_0076631 mediated pyroptosis in diabetic cardiomyopathy by functioning as a ceRNA, sponging endogenous miR-214-3p to sequester and inhibit its expression (Yang et al., 2019). For ischemic cardiomyopathy, circRNA circFndc3b was found to be significantly downregulated in cardiac tissues of ischemic cardiomyopathy patients, and overexpression of circRNA circFndc3b in cardiac endothelial cells increases vascular endothelial growth factor-A expression and enhances their angiogenic activity and reduces cardiomyocytes and endothelial cell apoptosis (Garikipati et al., 2019). Given the bioinformatic analysis results and ceRNA network, our study identified three circRNAs, namely, hsa_circ_0043762, has_circ_0036248, and has_circ_0071269, which might be key risk factors involved in HCM pathogenesis and highlighted their potential function in HCM.

Based on the GO functional annotations of circRNAs in the network, three of these circRNAs were enriched in calcium-release channel activity. As calcium homeostasis dysregulation is one of the most commonly described mechanisms, it is highly related to mutation-specific alterations in the rate of calcium release in HCM (Wu et al., 2019). Moreover, calcium homeostasis dysregulation might exacerbate diastolic dysfunction, which can progress into heart failure and lead to significant morbidity and mortality (Wu et al., 2019). Also, hsa_circ_0036248 was demonstrated to be associated with the TRP channel by KEGG analysis. TRP channels showed increased expression in the HCM model compared with the control group, which also likely contributed to diastolic calcium overload (Tang et al., 2019). Thus, this evidence strongly supports that the identified circRNAs in the ceRNA network may play important roles in the pathogenesis of HCM.

Although altered circRNAs and mRNAs were identified and their possible roles in the pathogenesis mechanisms of HCM were investigated, several limitations should be considered in interpreting our findings. Because of the limited samples and the lack of technical replicates, the power of the statistical conclusions might be limited, and it may not be powerful to conduct any subtype analysis. In our study, several databases were enrolled for the prediction of interactions between circRNAs and miRNAs and between mRNAs and miRNAs aiming to guarantee their reproducibility and reliability. In the future, with the emergence of larger sample sizes, better databases, and better algorithms, a more comprehensive ceRNA network will be constructed. Also, molecular biology methods, including qPCR, luciferase reporter systems, and coimmunoprecipitation assays, may help to validate our findings and may thus serve to unravel the molecular mechanisms of ceRNA networks in HCM.

## Conclusion

Our study constructed a circRNA-associated ceRNA network in HCM, and the identified circRNAs hsa_circ_0043762, hsa_circ_0036248, and hsa_circ_0071269 in the network may be key risk factors involved in HCM pathogenesis. Our study provides novel insight into the pathogenesis of HCM from the perspective of the circRNA–miRNA–mRNA network.

## Data Availability Statement

The datasets presented in this study can be found in online repositories. The names of the repository/repositories and accession number(s) can be found below: https://www.ncbi.nlm.nih.gov/geo/, GSE148602.

## Ethics Statement

The studies involving human participants were reviewed and approved by the Ethics Committee of Sun Yat-sen Memorial Hospital. The patients/participants provided their written informed consent to participate in this study.

## Author Contributions

QG and YZ conceived and designed the study. JJW and RS performed the experiments. MW, JB, and ZL analyzed the data. ZH, QC, and WL were responsible for the visualization of data. QG wrote the original draft of the manuscript. JFW and YZ supervised this work and revised the manuscript. All authors have read and approved the final manuscript.

## Conflict of Interest

The authors declare that the research was conducted in the absence of any commercial or financial relationships that could be construed as a potential conflict of interest.

## Supplementary Material

FIGURE S1 Box plot of original data and normalized data. **(A)** Box plot of original data. **(B)** Box plot of normalized data. The distributions of RNA intensity values of each sample were showed in box plot. Quantile algorithm was used for normalization of original data. HCM, hypertrophic cardiomyopathy.

FIGURE S2 Visualization of the gene network using a heatmap plot. The heatmap depicts the topological overlap matrix among circRNAs in the coexpression network analysis. The light color represents low overlap, and the progressively darker red color represents higher overlap. Blocks of darker colors along the diagonal are the modules. The gene dendrogram and module assignment are also shown along the left side and the top.

FIGURE S3 Scatterplots of MM vs. GS for clinical traits in key modules. **(A–C)** Scatterplots of MM in the magenta module vs. GS for HCM, thickness, obstruction, respectively. **(D–F)** Scatterplots of MM in red modules vs. GS for HCM, thickness, and obstruction, respectively. Correlation efficient and *p*-value were calculated and showed for each association. GS, gene significance; MM, module membership; HCM, hypertrophic cardiomyopathy; cor, correlation coefficient.

FIGURE S4 Functional annotation of circRNA host genes in key modules. **(A)** GO term terms for circRNA host genes in the magenta module. **(B)** KEGG terms for circRNA host genes in the magenta module. **(C)** GO terms for circRNA host genes in the red module. **(D)** KEGG terms for circRNA host genes in the red module. All *p*-values were adjusted by the Benjamini–Hochberg method. TOR, target of rapamycin; TGF, transforming growth factor; TCF, transcription factor.

FIGURE S5 Differential expression analysis of the circulating transcriptome by Limma. **(A)** Volcano plot for circRNAs. **(B)** Heatmap for differentially expressed circRNAs. **(C)** Volcano plot for mRNAs. **(D)** Heatmap for differentially expressed mRNAs. Each point in volcano plot corresponded to one RNA. Red points indicated upregulated RNAs and blue points indicated downregulated RNAs. A total of 391 upregulated circRNAs, 384 downregulated circRNAs, 229 upregulated mRNAs, and 140 downregulated mRNAs were identified by the Limma method. Both differentially expressed circRNAs and mRNAs can distinguish HCM patients and healthy controls. In this Limma analysis, the *p-value* was adjusted by the Benjamini–Hochberg method. HCM, hypertrophic cardiomyopathy; Limma, linear models for microarray data.

TABLE S1 Original intensity values of each RNA in each sample.

TABLE S2 Normalized intensity values of each RNA in each sample.

TABLE S3 Clinical characteristics of HCM group and control group.

TABLE S4 Hub differential expressed circRNAs in magenta module.

TABLE S5 Hub differential expressed circRNAs in red module.

TABLE S6 GO and KEGG terms for each circRNA in ceRNA network.
