# Supplementary figures and images for: Comprehensive Construction of a Circular RNA-Associated Competing Endogenous RNA Network Identified Novel Circular RNAs in Hypertrophic Cardiomyopathy by Integrated Analysis
Source: Front Genet. 2020 Jul 28;11:764. doi: 10.3389/fgene.2020.00764 (PMC7399352; doi:10.3389/fgene.2020.00764)

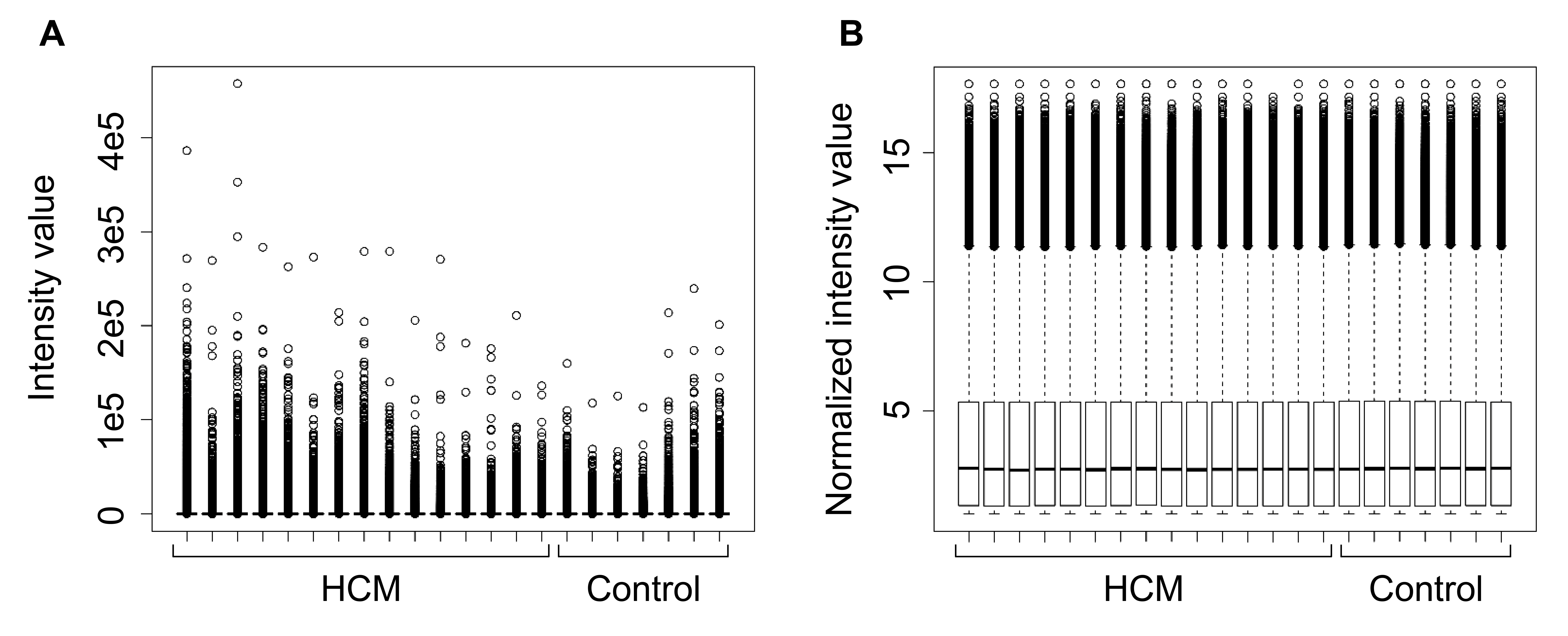

Supplement: FIGURE S1 — Box plot of original data and normalized data. (A) Box plot of original data. (B) Box plot of normalized data. The distributions of RNA intensity values of each sample were showed in box plot. Quantile algorithm was used for normalization of original data. HCM, hypertrophic cardiomyopathy. [file Image_1.TIF]

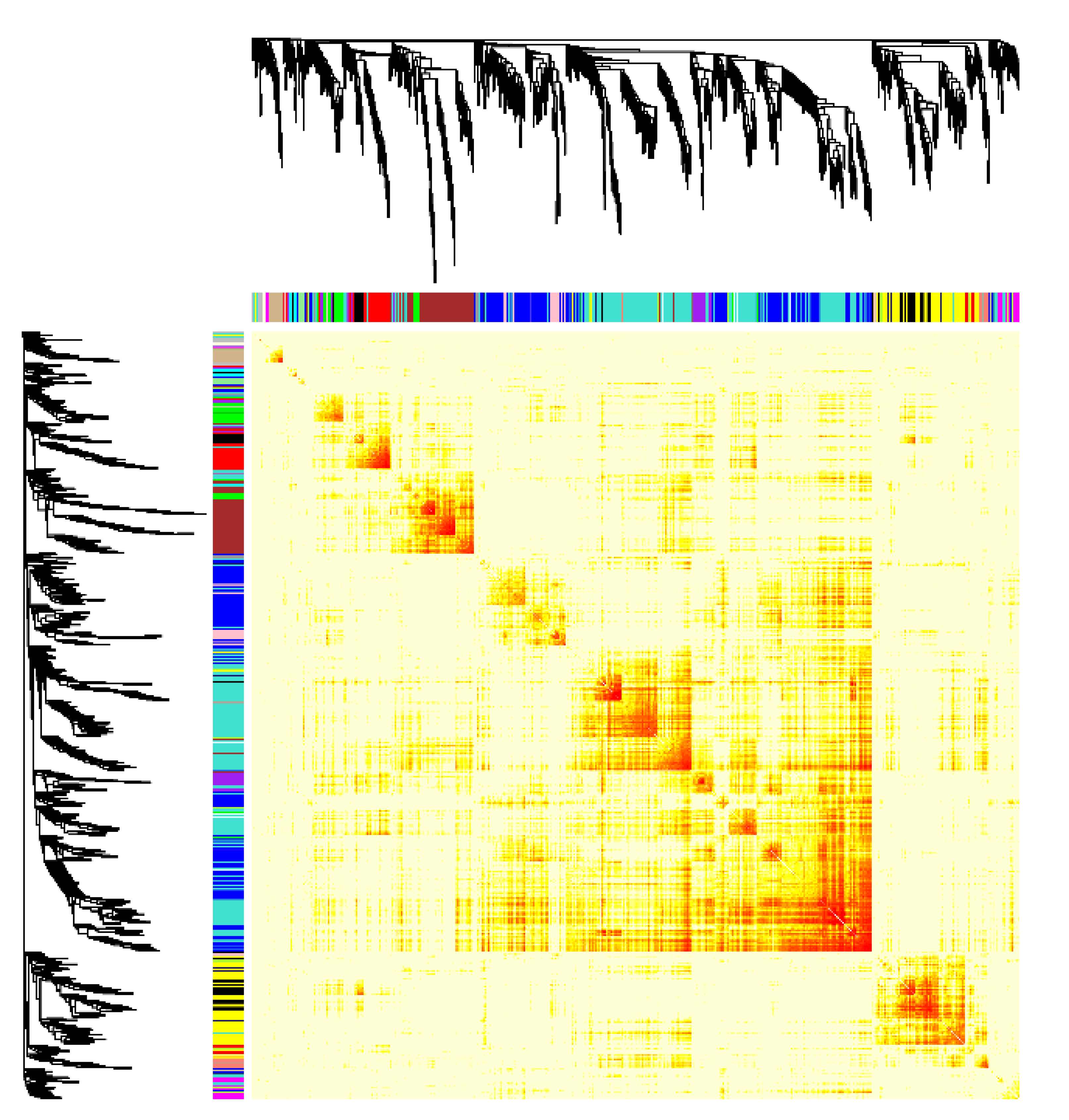

Supplement: FIGURE S2 — Visualization of the gene network using a heatmap plot. The heatmap depicts the topological overlap matrix among circRNAs in the coexpression network analysis. The light color represents low overlap, and the progressively darker red color represents higher overlap. Blocks of darker colors along the diagonal are the modules. The gene dendrogram and module assignment are also shown along the left side and the top. [file Image_2.TIF]

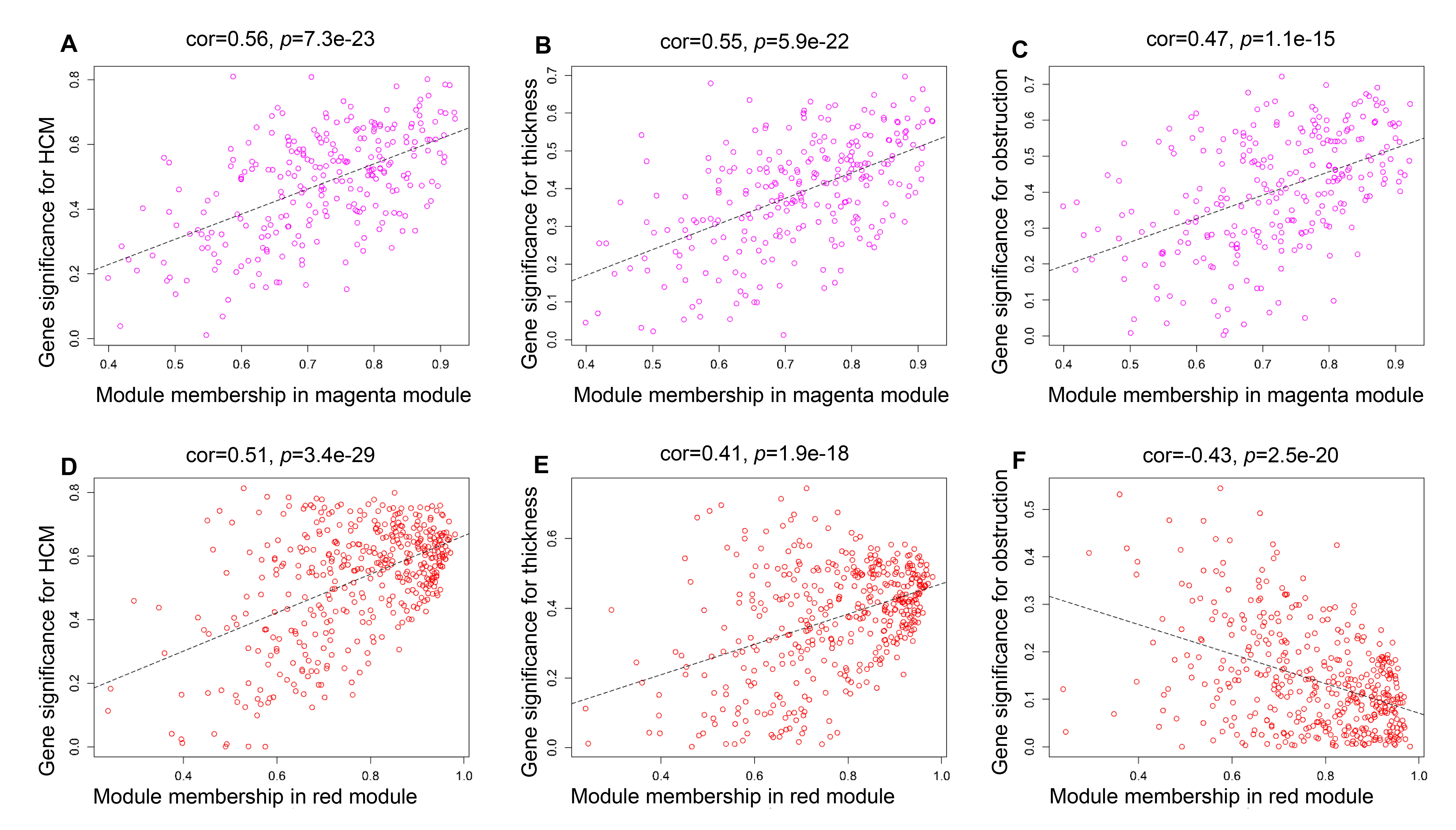

Supplement: FIGURE S3 — Scatterplots of MM vs. GS for clinical traits in key modules. (A–C) Scatterplots of MM in the magenta module vs. GS for HCM, thickness, obstruction, respectively. (D–F) Scatterplots of MM in red modules vs. GS for HCM, thickness, and obstruction, respectively. Correlation efficient and p-value were calculated and showed for each association. GS, gene significance; MM, module membership; HCM, hypertrophic cardiomyopathy; cor, correlation coefficient. [file Image_3.TIF]

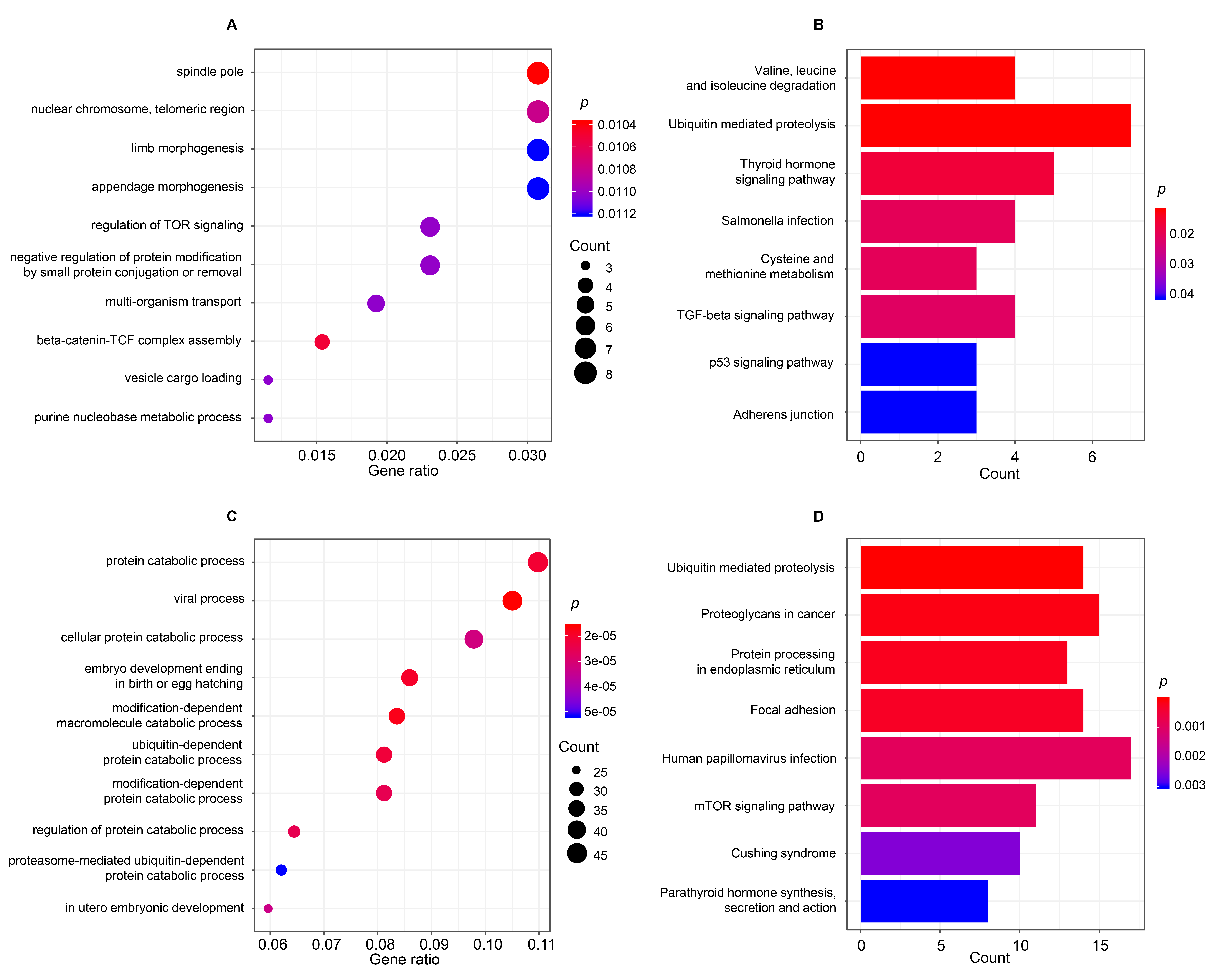

Supplement: FIGURE S4 — Functional annotation of circRNA host genes in key modules. (A) GO term terms for circRNA host genes in the magenta module. (B) KEGG terms for circRNA host genes in the magenta module. (C) GO terms for circRNA host genes in the red module. (D) KEGG terms for circRNA host genes in the red module. All p-values were adjusted by the Benjamini–Hochberg method. TOR, target of rapamycin; TGF, transforming growth factor; TCF, transcription factor. [file Image_4.TIF]

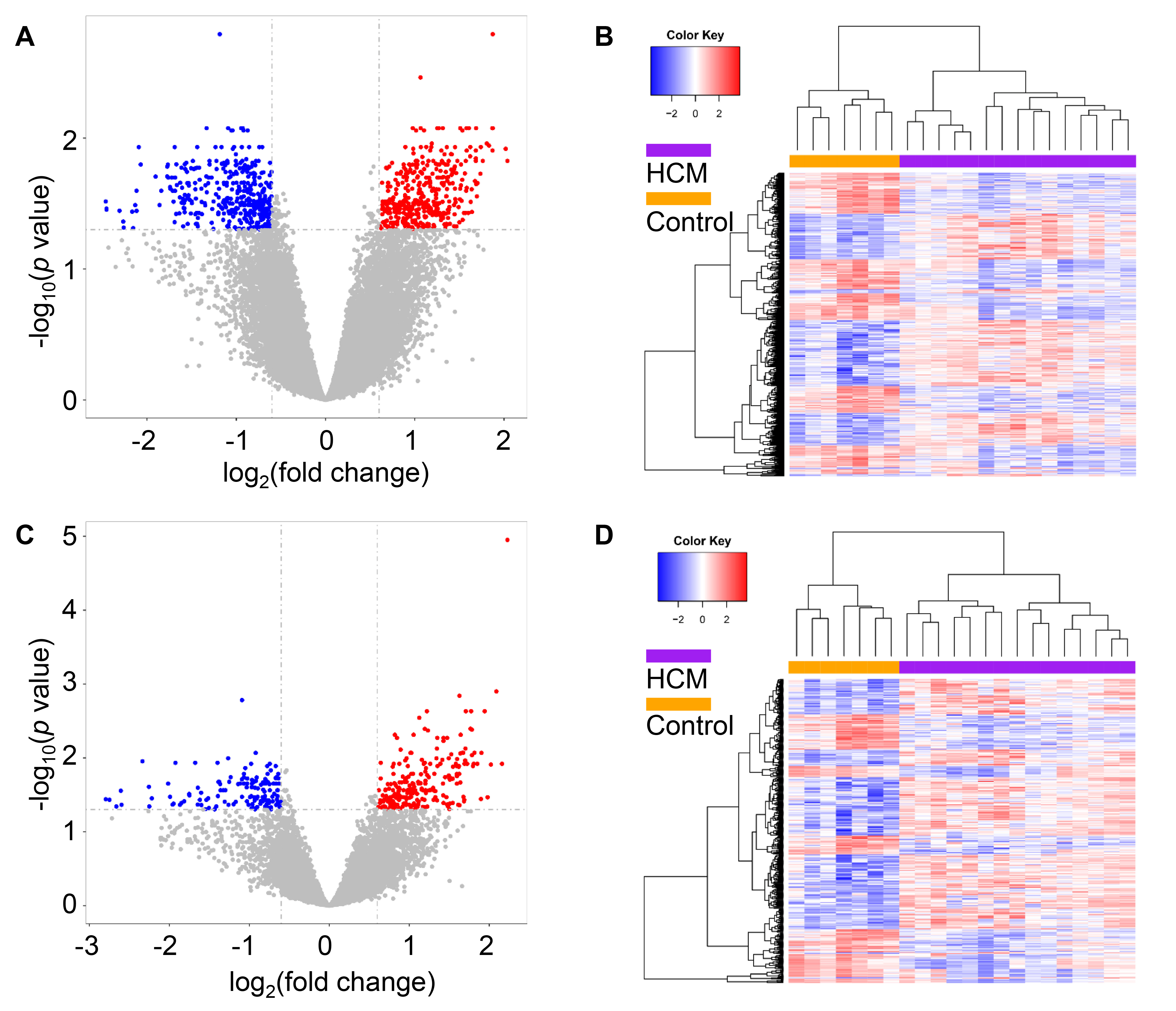

Supplement: FIGURE S5 — Differential expression analysis of the circulating transcriptome by Limma. (A) Volcano plot for circRNAs. (B) Heatmap for differentially expressed circRNAs. (C) Volcano plot for mRNAs. (D) Heatmap for differentially expressed mRNAs. Each point in volcano plot corresponded to one RNA. Red points indicated upregulated RNAs and blue points indicated downregulated RNAs. A total of 391 upregulated circRNAs, 384 downregulated circRNAs, 229 upregulated mRNAs, and 140 downregulated mRNAs were identified by the Limma method. Both differentially expressed circRNAs and mRNAs can distinguish HCM patients and healthy controls. In this Limma analysis, the p-value was adjusted by the Benjamini–Hochberg method. HCM, hypertrophic cardiomyopathy; Limma, linear models for microarray data. [file Image_5.TIF]
