# Supplementary material for: Comprehensive Construction of a Circular RNA-Associated Competing Endogenous RNA Network Identified Novel Circular RNAs in Hypertrophic Cardiomyopathy by Integrated Analysis
Source: Front Genet. 2020 Jul 28;11:764. doi: 10.3389/fgene.2020.00764 (PMC7399352; doi:10.3389/fgene.2020.00764)
Supplement: TABLE S1 — Original intensity values of each RNA in each sample. [file Data_Sheet_1.zip › Supplementary Table 3 - 6.DOC]

Supplementary Table S3 Clinical characteristics of HCM group and control group

| **Characteristics** | **Hypertrophic cardiomyopathy** | **Controls** | ***P* values** |
| --- | --- | --- | --- |
| Subjects, n | 15 | 7 |  |
| Age, years | 59.26±11.81 | 54.71±7.49 | 0.364 |
| Males, n(%) | 8 (53.33) | 3 (42.85) | 0.647 |
| BMI, kg/m^2^ | 23.85±2.71 | 22.36±1.39 | 0.189 |
| Smoking, n(%) | 5 (33.33) | 0 (0.00) | 0.135 |
| Total cholesterol, mmol/L | 4.21±0.71 | 6.03±1.20 | ＜0.001 |
| Triglyceride, mmol/L | 1.44±0.92 | 1.08±0.50 | 0.350 |
| White blood cell count, ×10^9^/L | 7.41±1.16 | 6.24±0.91 | 0.029 |
| Blood glucose, mmol/L | 4.67±0.64 | 5.40±0.61 | 0.022 |
| Hemoglobin, g/L | 137.46±15.90 | 143.57±11.08 | 0.373 |
| Creatinine, μmol/L | 83.80±13.65 | 72.85±16.74 | 0.118 |
| Systolic blood pressure, mmHg | 122.40±9.61 | 116.57±8.46 | 0.185 |
| Diastolic blood pressure, mmHg | 72.20±12.18 | 78.57±9.14 | 0.235 |
| LVEF, % | 68.06±8.70 | 67.71±6.92 | 0.926 |
| LVDd, mm | 45.46±6.90 | 45.42±4.27 | 0.989 |
| Maximum LVWT, mm | 19.86±4.38 | 9.28±1.25 | ＜0.001 |
| LA, mm | 39.00±6.03 | 31.71±2.36 | 0.006 |
| ICD, n(%) | 8 (53.33) | 0 (0.00) | 0.022 |

Categorical parameters were presented as n(%), and continuous parameters were presented as mean±SD. BMI, body-mass index; HCM, hypertrophic cardiomyopathy; ICD, implantable cardioverter-defibrillator; LVEF, left ventricular ejection fraction; LVWT, left ventricular wall thickness; LVDd, left ventricular diastolic dimension; LA, left atrial diameter.

Supplementary Table S4 Hub differential expressed circRNAs in magenta module

| Number | circRNA | log_2_FC | *p* value | Gene symbol |
| --- | --- | --- | --- | --- |
| 1 | hsa_circ_0007933 | 1.87 | 0.008 | MID1 |
| 2 | hsa_circ_0063629 | 1.31 | 0.008 | CENPM |
| 3 | hsa_circ_0064012 | 1.45 | 0.012 | CHKB-CPT1B |
| 4 | hsa_circ_0010662 | 1.18 | 0.012 | HSPG2 |
| 5 | hsa_circ_0069693 | 1.13 | 0.012 | FRYL |
| 6 | hsa_circ_0007718 | 1.58 | 0.012 | MID1 |
| 7 | hsa_circ_0052587 | 1.41 | 0.013 | RRM2 |
| 8 | hsa_circ_0072846 | 1.21 | 0.015 | *NA* |
| 9 | hsa_circ_0066918 | 1.52 | 0.016 | HGD |
| 10 | hsa_circ_0061440 | 1.43 | 0.018 | URB1 |
| 11 | hsa_circ_0012687 | 1.56 | 0.019 | USP24 |
| 12 | hsa_circ_0085207 | 1.74 | 0.019 | UBR5 |
| 13 | hsa_circ_0039481 | 1.03 | 0.020 | NUP93 |
| 14 | hsa_circ_0071854 | 1.31 | 0.020 | MARCH6 |
| 15 | hsa_circ_0041336 | 1.16 | 0.020 | SERPINF2 |
| 16 | hsa_circ_0058150 | 1.17 | 0.021 | FN1 |
| 17 | hsa_circ_0070281 | 1.04 | 0.023 | AGPAT9 |
| 18 | hsa_circ_0068363 | 1.45 | 0.024 | VPS8 |
| 19 | hsa_circ_0069553 | 1.08 | 0.026 | RBM47 |
| 20 | hsa_circ_0034290 | 1.24 | 0.027 | TJP1 |
| 21 | hsa_circ_0089916 | 1.08 | 0.027 | PIGA |
| 22 | hsa_circ_0036314 | 1.24 | 0.027 | FAM219B |
| 23 | hsa_circ_0045900 | 1.36 | 0.028 | PGS1 |
| 24 | hsa_circ_0043058 | 1.01 | 0.030 | MYO1D |
| 25 | hsa_circ_0043059 | 1.39 | 0.030 | MYO1D |
| 26 | hsa_circ_0002415 | 1.28 | 0.031 | MID1 |
| 27 | hsa_circ_0067932 | 1.18 | 0.034 | PRKCI |
| 28 | hsa_circ_0040531 | 1.24 | 0.034 | WDR59 |
| 29 | hsa_circ_0023984 | 1.15 | 0.036 | NOX4 |
| 30 | hsa_circ_0077738 | 1.28 | 0.037 | CEP85L |
| 31 | hsa_circ_0066285 | 1.18 | 0.038 | SLMAP |
| 32 | hsa_circ_0000060 | 1.36 | 0.046 | *NA* |
| 33 | hsa_circ_0018484 | 1.22 | 0.047 | HERC4 |
| 34 | hsa_circ_0081256 | 1.07 | 0.049 | TRRAP |
| 35 | hsa_circ_0043065 | 1.04 | 0.049 | MYO1D |

A total of 35 hub differential expressed circRNAs were identified in magenta module. In this current analysis, *p* value was adjusted by Benjamini-Hochberg method. NA, not available; circRNA, circular RNA.

Supplementary Table S5 Hub differential expressed circRNAs in red module

| Number | circRNA | log_2_FC | *p* value | Gene symbol |
| --- | --- | --- | --- | --- |
| 1 | hsa_circ_0026850 | 1.87 | 0.008 | ESYT1 |
| 2 | hsa_circ_0090676 | 1.69 | 0.008 | HUWE1 |
| 3 | hsa_circ_0043477 | 1.81 | 0.011 | GRB7 |
| 4 | hsa_circ_0053657 | 1.36 | 0.012 | BIRC6 |
| 5 | hsa_circ_0074252 | 1.62 | 0.012 | ANKHD1-EIF4EBP3 |
| 6 | hsa_circ_0078450 | 2.02 | 0.012 | SYNJ2 |
| 7 | hsa_circ_0087926 | 1.87 | 0.012 | TMEM245 |
| 8 | hsa_circ_0007171 | 1.62 | 0.014 | MYO9B |
| 9 | hsa_circ_0036450 | 1.76 | 0.015 | WDR61 |
| 10 | hsa_circ_0033893 | 2.04 | 0.015 | *NA* |
| 11 | hsa_circ_0083938 | 1.32 | 0.015 | GPR124 |
| 12 | hsa_circ_0071269 | 1.09 | 0.015 | FBXW7 |
| 13 | hsa_circ_0087707 | 1.35 | 0.016 | TRIM14 |
| 14 | hsa_circ_0023937 | 1.14 | 0.016 | PICALM |
| 15 | hsa_circ_0058690 | 1.45 | 0.017 | DIS3L2 |
| 16 | hsa_circ_0015161 | 1.06 | 0.018 | BLZF1 |
| 17 | hsa_circ_0053664 | 1.41 | 0.019 | BIRC6 |
| 18 | hsa_circ_0036248 | 1.38 | 0.020 | ADPGK |
| 19 | hsa_circ_0038633 | 1.36 | 0.020 | PLK1 |
| 20 | hsa_circ_0062517 | 1.13 | 0.020 | RAB36 |
| 21 | hsa_circ_0002101 | 1.67 | 0.021 | LATS1 |
| 22 | hsa_circ_0026496 | 1.15 | 0.022 | EIF4B |
| 23 | hsa_circ_0048407 | 1.06 | 0.022 | SPPL2B |
| 24 | hsa_circ_0017263 | 1.17 | 0.022 | ADSS |
| 25 | hsa_circ_0044368 | 1.42 | 0.022 | ATP5G1 |
| 26 | hsa_circ_0006815 | 1.39 | 0.022 | WNT5A |
| 27 | hsa_circ_0005507 | 1.51 | 0.024 | CRIM1 |
| 28 | hsa_circ_0028611 | 1.36 | 0.024 | FBXW8 |
| 29 | hsa_circ_0055719 | 1.27 | 0.025 | FAHD2B |
| 30 | hsa_circ_0052574 | 1.22 | 0.025 | TAF1B |
| 31 | hsa_circ_0031738 | 1.05 | 0.025 | TRAPPC6B |
| 32 | hsa_circ_0076133 | 1.22 | 0.025 | FANCE |
| 33 | hsa_circ_0074352 | 1.58 | 0.025 | PCDH1 |
| 34 | hsa_circ_0079943 | 1.19 | 0.025 | SUGCT |
| 35 | hsa_circ_0041499 | 1.04 | 0.026 | ZZEF1 |
| 36 | hsa_circ_0021190 | 1.07 | 0.029 | IPO7 |
| 37 | hsa_circ_0009077 | 1.50 | 0.029 | TYW1B |
| 38 | hsa_circ_0060045 | 1.15 | 0.030 | EDEM2 |
| 39 | hsa_circ_0041226 | 1.00 | 0.030 | MYO1C |
| 40 | hsa_circ_0032278 | 1.53 | 0.030 | RDH11 |
| 41 | hsa_circ_0043762 | 1.08 | 0.030 | KAT2A |
| 42 | hsa_circ_0020795 | 1.46 | 0.031 | TSPAN32 |
| 43 | hsa_circ_0009854 | 1.12 | 0.031 | AGTRAP |
| 44 | hsa_circ_0057891 | 0.98 | 0.031 | PARD3B |
| 45 | hsa_circ_0003524 | 1.09 | 0.032 | FAM168B |
| 46 | hsa_circ_0053667 | 1.49 | 0.034 | BIRC6 |
| 47 | hsa_circ_0056476 | 0.96 | 0.034 | FAM168B |
| 48 | hsa_circ_0079157 | 1.16 | 0.034 | SNX8 |
| 49 | hsa_circ_0048229 | 1.10 | 0.034 | DAZAP1 |
| 50 | hsa_circ_0053673 | 1.14 | 0.034 | BIRC6 |
| 51 | hsa_circ_0062002 | 0.94 | 0.034 | ADARB1 |
| 52 | hsa_circ_0018419 | 1.26 | 0.035 | BICC1 |
| 53 | hsa_circ_0077741 | 0.97 | 0.035 | MAN1A1 |
| 54 | hsa_circ_0041881 | 1.37 | 0.035 | POLR2A |
| 55 | hsa_circ_0091191 | 1.02 | 0.036 | POF1B |
| 56 | hsa_circ_0022238 | 1.18 | 0.036 | VWCE |
| 57 | hsa_circ_0075992 | 1.11 | 0.036 | ITPR3 |
| 58 | hsa_circ_0022557 | 1.64 | 0.037 | SNHG1 |
| 59 | hsa_circ_0065678 | 1.03 | 0.037 | APEH |
| 60 | hsa_circ_0022958 | 1.37 | 0.037 | PACS1 |
| 61 | hsa_circ_0010999 | 1.17 | 0.037 | DHDDS |
| 62 | hsa_circ_0076231 | 0.95 | 0.037 | CMTR1 |
| 63 | hsa_circ_0054592 | 1.55 | 0.037 | EML6 |
| 64 | hsa_circ_0041554 | 1.17 | 0.037 | UBE2G1 |
| 65 | hsa_circ_0007366 | 1.35 | 0.038 | ANK1 |
| 66 | hsa_circ_0053882 | 1.04 | 0.038 | TTC27 |
| 67 | hsa_circ_0088148 | 1.02 | 0.038 | RGS3 |
| 68 | hsa_circ_0047957 | 1.26 | 0.038 | CNDP2 |
| 69 | hsa_circ_0037515 | 1.11 | 0.040 | ABCA3 |
| 70 | hsa_circ_0047285 | 1.13 | 0.040 | TTC39C |
| 71 | hsa_circ_0019508 | 1.29 | 0.040 | CWF19L1 |
| 72 | hsa_circ_0038565 | 1.12 | 0.040 | COG7 |
| 73 | hsa_circ_0040422 | 1.01 | 0.042 | DHX38 |
| 74 | hsa_circ_0024795 | 0.97 | 0.044 | CHEK1 |
| 75 | hsa_circ_0030913 | 1.39 | 0.045 | ARHGEF7 |
| 76 | hsa_circ_0007685 | 1.09 | 0.046 | WWC3 |
| 77 | hsa_circ_0064946 | 1.03 | 0.046 | CTNNB1 |
| 78 | hsa_circ_0016664 | 1.02 | 0.046 | SRP9 |
| 79 | hsa_circ_0025665 | 1.21 | 0.046 | ITPR2 |
| 80 | hsa_circ_0076429 | 1.15 | 0.047 | PEX6 |
| 81 | hsa_circ_0032281 | 1.44 | 0.047 | RDH11 |
| 82 | hsa_circ_0006630 | 1.04 | 0.048 | *NA* |
| 83 | hsa_circ_0011555 | 1.09 | 0.049 | ZMYM4 |

A total of 83 hub differential expressed circRNAs were identified in red module. In this current analysis, *p* value was adjusted by Benjamini-Hochberg method. NA, not available; circRNA, circular RNA.

Supplementary Table S6 GO and KEGG terms for each circRNA in ceRNA network

| circRNA  (host gene) | Fold change | *p* |  | GO term |  | KEGG term |
| --- | --- | --- | --- | --- | --- | --- |
| hsa_circ_0036248  (ADPGK) | 2.59 | 0.020 |  | GO:0030049 muscle filament sliding  GO:0033275 actin-myosin filament sliding  GO:0043534 blood vessel endothelial cell migration  GO:0006880 intracellular sequestering of iron ion  GO:0015278 calcium-release channel activity |  | KEGG:04750 Inflammatory mediator regulation of TRP channels  KEGG:04261 Adrenergic signaling in cardiomyocytes  KEGG:04020 Calcium signaling pathway  KEGG:04064 NF-kappa B signaling pathway  KEGG:04924 Renin secretion |
| hsa_circ_0041554  (UBE2G1) | 2.25 | 0.037 |  | GO:0002576 platelet degranulation  GO:0033212 iron import into cell  GO:0070491 repressing transcription factor binding |  |  |
| hsa_circ_0071269  (FBXW7) | 2.12 | 0.015 |  | GO:0043534 blood vessel endothelial cell migration  GO:0035050 embryonic heart tube development  GO:0046716 muscle cell cellular homeostasis  GO:0051282 regulation of sequestering of calcium ion  GO:0015278 calcium-release channel activity |  | KEGG:05416 Viral myocarditis  KEGG:04750 Inflammatory mediator regulation of TRP channels  KEGG:04530 Tight junction  KEGG:04940 Type I diabetes mellitus  KEGG:05414 Dilated cardiomyopathy |
| hsa_circ_0011555  (ZMYM4) | 2.12 | 0.049 |  | GO:0002576 platelet degranulation  GO:0033212 iron import into cell  GO:0070491 repressing transcription factor binding |  |  |
| hsa_circ_0043762  (KAT2A) | 2.10 | 0.030 |  | GO:0015278 calcium-release channel activity  GO:0016529 sarcoplasmic reticulum  GO:1990928 response to amino acid starvation  GO:0019674 NAD metabolic process  GO:0016860 intramolecular oxidoreductase activity |  | KEGG:05120 Epithelial cell signaling in Helicobacter pylori infection  KEGG:04662 B cell receptor signaling pathway |
| hsa_circ_0041499  (ZZEF1) | 2.05 | 0.026 |  | GO:0043403 skeletal muscle tissue regeneration  GO:0019674 NAD metabolic process  GO:0090077 foam cell differentiation  GO:0016860 intramolecular oxidoreductase activity  GO:0002576 platelet degranulation |  | KEGG:04640 Hematopoietic cell lineage  KEGG:05120 Epithelial cell signaling in Helicobacter pylori infection  KEGG:05203 Viral carcinogenesis |

In this current analysis, *p* value was adjusted by Benjamini-Hochberg method. CircRNA, circular RNA; ceRNA, competing endogenous RNA; GO, gene ontology; KEGG, kyoto encyclopedia of genes and genomes; NAD, nicotinamide adenine dinucleotide; TRP, transient receptor potential.
